# Population health management genomic new-born screens and multi-omics intercepts

**DOI:** 10.3389/frai.2024.1496942

**Published:** 2025-08-01

**Authors:** James Andrew Henry

**Affiliations:** Institute of Biomedical Sciences, London, United Kingdom

**Keywords:** genomic new-born screens, predictive health pre-eXams, precision care eXams, human phenotype ontology, AI digital regulation service

## Abstract

**Introduction:**

The Population Health Management (PHM) Genomic Newborn Screens (GNBS) and Multi-Omics Intercepts for Human Phenotype Ontology (HPO) using Federated Data Platforms (FDP) represent a groundbreaking innovation in global health. This reform, supported by the UK’s Genomic Medical Services (GMS) through “The Generation Study,” aims to significantly reduce infant mortality by identifying and managing over 200 rare diseases from birth, paving the way for personalised health planning.

**Methods:**

Using an ecosystem approach, this study evaluates a diverse pangenome to predict health outcomes or confirm diagnoses prior to symptomatic manifestations. GNBS standardises care by integrating diagnostic techniques such as blood spot analysis and full blood cell diagnostics to stratify risk. The approach enhances the understanding of rare diseases in primary care medicine, with biomedical and haematology diagnoses re-evaluated. Scientific proof of concept and fit-for-purpose technology align multi-omics in pre-eXams (X = Gen AI).

**Recommendations:**

The Digital Regulation Service (DRS) assembles an agile group of experts to enhance medical science through human phenotype ontology (HPO) for precise disease segmentation, scheduling accurate eXam intercepts where needed. This team strategically plans regulation services for digital HPO eXam assurance and implements Higher Expert Medical Science Safety (HEMSS) frameworks. The DRS is responsible for overseeing gene, oligonucleotide, and recombinant protein intercepts; commissioning blood pathology HPO eXam intercepts; and monitoring preliminary eXams with advanced imaging techniques.

**Discussion:**

In pursuit of excellence in PHM of HPO, HEMSS with Agile Group Development leverages the Genomic Newborn Screens (GNBS) and multi-omics to create personalised health plans integrated with NHS England Genomics and AI-driven DRS. The discourse extends to examining GNBS predictors and intercepts, focusing on their impact on public health and patient safety. Discussions encompass structured HPO knowledge addressing newborn health, ethical considerations, family privacy, and the benefits and limitations of pre-eXam screenings and life eXam intercepts. These debates involve stakeholders in adopting HPO-enhanced clinical pathways through Alliances for Health Systems Networking-Genomic Enterprise Partnerships (AHSN-GEP).

**Conclusion:**

“The Generation Study” represents a paradigm in digital child health management using an HPO-X-Gen-AI framework, transitioning from trusted research to evidence-based discovery. This approach sets a standard for personalised healthcare practices, incorporating ontology risk stratification and future-ready analytics as outlined in the NHS Constitution. The discourse on higher expert medical science safety governance will continue in the forthcoming manuscript, “PHM Fit Lifecycles in Future Analytics,” which will further explore developing localised health solutions for “Our Future Health.”

## 1 Introduction to genomic newborn screens for a national programme

The World Health Organisation reported the high rates of preventable death and poor health and later issued principles for collecting, accessing, using, and sharing genomic data to improve wellbeing (World Health Organisation, 2023; World Health Organisation, 2024). Five years have passed since the UK National Screening Committee and Genomics England reported on the implications of Genome Newborn Screening (GNBS), which is capable of accurately diagnosing a broad spectrum of rare diseases and genetic conditions at birth (UK National Screening Committee, 2023; Jiang et al., 2023) As the Lancet Journal informs of GNBS false negatives, the Nature Journal advocates accurate genomics to screen lifecycles (Lancet, 2023; Stark and Scott, 2023).

**Figure 1:**
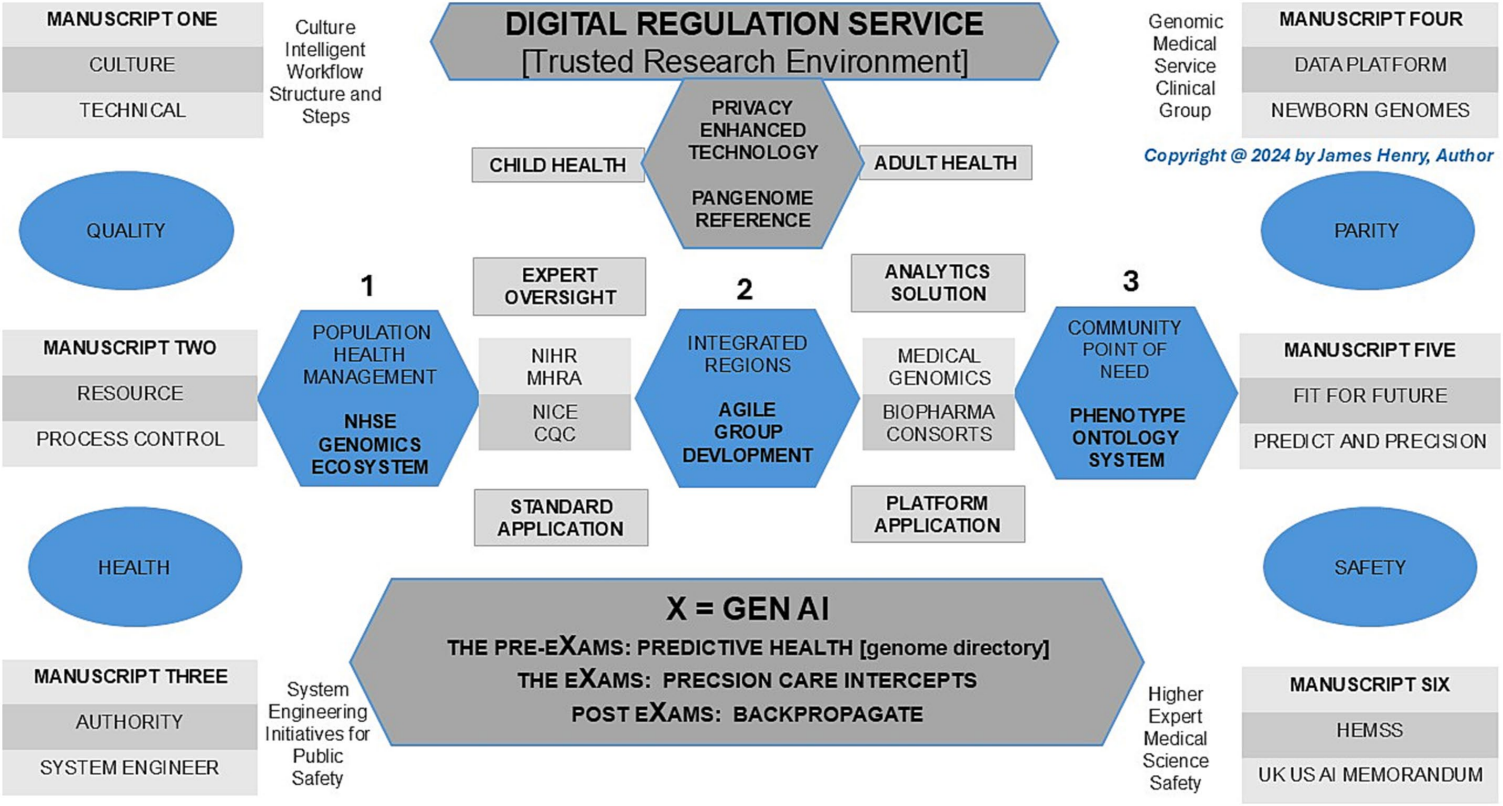
Population health management, genomics newborn screens, and multi-omics with intercepts as an ecosystem.

In 2025, “The Generation Study” will have recruited 100 K newborns for Whole Genome Sequencing (WGS) to predict over 200 rare diseases using a new pangenome reference for a lifecycle of accurate intercepts (NHS England, 2024; Liao et al., 2023). This article for “Genomics Newborn Screen with Multi-Omics Intercepts” is number four in a series titled “Population Health Management Higher Expert Medical Science Safety for Agile Group Development” as a proposal for public health, patient safety, and parity. My introduction to GNBS is that they predict health in pre-eXams, which scope precise care in eXams (X = Gen AI) (View Figure 1).

In Figure 1, Population Health Management (PHM) of Human Phenotype Ontology (HPO) in a lifetime is realised in a safe space, as depicted under the umbrella of the AI Digital Regulation Service (AIDRS) when aligned with the author’s programme proposal to accelerate NHS England (NHSE) Genomics strategy with AI infrastructure capacity (NHS Beta, 2024; NHSE, 2024; NHS England, 2022). Following the illustration, the paper first addresses the PHM and manuscript overview while providing a history of newborn blood spot screens and blood cell diagnostics to evidence the value of GNBS in the programme proposal. Moreover, a background on Federated Data Platforms (FDP) and their impact on primary care provides insights into the ecosystem advantage. These platforms enhance our ability to integrate multi-omics data and intercepts for the PHM of each HPO.

## 2 Population health management and manuscript overview

In a PHM overview, this programme pioneers the landscape for HPO with AI digital partners and healthcare providers analysing vast amounts of data to predict health trends, personalise care plans, and continually improve ecosystems through backpropagation (van Ede et al., 2023). As more data is collected in an ecosystem, there is more accurate risk stratification, which we learn from Trusted Research Environments (TRE) for evidence in HPO disease segmentation, providing increasingly precise and effective health interventions to deploy the programme for NHSE Genomics (Kerasidou et al., 2023).

Section 3 traces the history of newborn blood spot screens, from traditional mass spectrometry for conditions like phenylketonuria to the enhanced prediction of modern Genomic Newborn Screens. The author introduces a wider range of disorders from alternate perspective, like hematology, in which genomics panels evolve in modeling for a more effective and accurate diagnosis or prediction. Section 4 provides a background on federated data platforms (FDPs), highlighting the lessons learned from previous data-sharing initiatives like UK Care Data, and discusses how new FDPs, with robust stewardship, can securely share genetic information for research and precision medicine while complying with data protection regulations.

Section 5 introduces the science and technology behind child digital ontology, focusing on its aims for lifetime predictors and intercepts. Section 6 reviews the updated pangenome version and discusses genome pre-eXams while assessing the efficacy of traditional newborn blood spot tests and haematological blood cell diagnostics for rapid, effective diagnosis. Section 7 details the actions of the artificial intelligence-driven regulatory science of predictive pre-eXam development and eXam intercept adoption that phase timely and accurate biopharmaceutical therapies. Section 8 discusses the HPO as a system, engaging stakeholders on the ethics, benefits, limitations, and assurances provided by federated data platforms (FDP). Section 9 concludes with PHM HPO X in the context of a pilot proposal for Higher Expert Medical Science Safety (HEMSS) with agile development.

## 3 History of newborn blood spot screens and blood cell diagnostics

The rare disease phenylketonuria gave rise to a newborn biomedical blood spot panel using mass spectrometry, a method with false negatives with infant morbidity likely (Følling, 1994; Clague and Thomas, 2002; O’Connor et al., 2018). A global rare disease spectrum has value in universal GNBS to identify thousands of diseases (Haendel et al., 2020; Kingsmore et al., 2022). GNBS with data sharing represents a major transformation in newborn screening. Unlike standard biomedical blood spot tests that analyze about 10 diseases at birth, this new approach brings greater significance to patient safety and public health opportunities (Taruscio and Gahl, 2024; Clague and Thomas, 2002). Nevertheless an ecosystem that risk stratifies untreatable disorders or where disorders do not respond to treatment is also contentious (Zhang et al., 2019).

Traditional haematology often enquires, “What’s new?” and extends invitations to contribute to special editions. While morphological and haematological analyses are effective, they often lack the precision for early and accurate diagnosis (Up to Date, 2019; Urrechaga, 2024). HPO enhances genome translation for differential blood cell, coagulation, and hemato-oncology predictors, providing significant value in early intercepts for diagnosis (Provan and Lazarus, 2024; Duncavage et al., 2022). The integration of Genomic Newborn Screens (GNBS) streamlines the diagnostic process, significantly reducing time, resource use, and follow-up requirements. With GNBS, diagnoses of biomedical and haematological disorders are enhanced, allowing Artificial Intelligence-Driven Regulatory Science to stratify HPO risks and group disorders effectively. This process facilitates agile intercepts using an NHS England genomics directory and machine learning/artificial intelligence (ML/AI) to propose therapies (Office, 2023), as depicted in Figure 1.

## 4 Background to federated data platforms

A background to previous UK Care Data use informed of insecurities while pandemic plans shared data for digital health to save lives (Limb, 2016; NHS Digital, 2023). Value in “What can we salvage from care data?” is trusting in GNBS opportunities with child digital health as a secure ontology system (Godlee, 2016; Bick et al., 2022). Biobank Agile Group developments require the FDP to share and store valuable genetic information for ongoing research to translate HPO for routine and evidence-based digital personalised plans (Alvarellos et al., 2023). The Royal College of Pathologists and the Institute of Biomedical Sciences aim to cycle digital health and justify getting the HPO right in striving to meet patients’ needs with predictive health and precision medicine through confidential data sharing (Royal College of Pathologists, 2022; Get It Right First Time, 2022).

Rebuking concerns that showcase the FDP allows stakeholders to reform primary care with genomic screens (Independent, 2023). Corporate governance assures data privacy on FDP use, and data privacy regulation requirements are firmly met (National Data Guardian, 2023; GOV.UK, 2018; European Union, 2018). PHM requires FDPs to secure citizens’ details and HPO data while compliant with the transition to the Data Protection and Digital Information Bill (GOV.UK, 2023c). The Department of Science, Innovation, and Technology (DSIT) and the AI Safety Institute (AISI) are involved in data security and algorithm assurance (GOV.UK, 2024). PHM across the FDP for HPO is an agile process to predict and diagnose health in pre-eXams with precise eXam intercepts strategically deployed at the point of need (Institute of Biomedical Sciences, 2023).

## 5 Science and technology aims for child digital health records

Figure 1 adopts a systems approach involving GMS providers and cloud hybrid digital partners to establish a blueprint for health and wellbeing. The UK Government Science and Technology Strategy mandates this growth strategy with a systematic organisation to reduce NHSE negligence claims, which peaked at £2.7 billion (Department of Science, Innovation and Technology, 2023; Dyer, 2023).

It outlines the structure for Child Digital Health Records in Section 5.1, Science and Technology Strategy; Section 5.2, Genomic Medical Service Ecosystem Providers; and Section 5.3, Cloud Hybrid Digital System Partners.

### 5.1 Science and technology strategy

The robust and repeatable approach to identifying technologies for PHM, as set forth by AISI and GMS, aims to engineer biology using Google, DeepMind, and OpenAI servers (GOV.UK, 2024; Department of Science, Innovation and Technology, 2023). The AIDRS develops infrastructure for the PHM projects to and from an FDP for health providers and communities to adopt an HPO system approach (NHS Beta, 2024; NHSE, 2024). In a confederation for GNBS studies, Populace Health provides insights into the “Our Future Health” programme (NHS Confederation, 2023; Our Future Health, 2021) as the US aligns “All of Us” and “EU4Health” also monitor UK progress (National Institutes of Health, 2019; European Commission, 2021).

Within the UK Science and Technology Strategy, the “robust and repeatable” has a caveat since general-purpose AI in a digital reform negatively impacts the PHM continuity at the point of need (Department of Science, Innovation and Technology, 2023; Coile, 2024). A GMS ecosystem that supports GNBS may later unlock adult DNA passports and other alliance determinants with cloud hybrid digital systems for our points of need (GOV.UK, 2023a; UK Health Data Research Alliance, 2024; Institute of Biomedical Sciences, 2023). The DSIT accounts for a GMS ecosystem approach with cloud hybrid systems as the author develops HPO systems as a primary care pinnacle to confederate public health and patient safety (NHS Confederation PCN Network, 2022).

### 5.2 Genomic medical service ecosystem providers

In a PHM ecosystem, NHS GMS technology transforms health with WGS for GNBS as the primary care, enabling quicker diagnoses, personalised treatments, and improved patient outcomes, while Genomics England provides a national directory for safe HPO space (Office, 2023; NHS England, 2017). An ecosystem national assurance on genome tests directs to the HPO dimension as cloud hybrid partners with agile groups develop PHM (Office, 2023). Therin GNBS predicts rare diseases and conditions we consider “major” in our national frameworks and strategies (GOV.UK, 2021a; GOV.UK, 2023b).

Figure 1 conveys the ecosystem for people in an NHS workforce plan who benefit from Higher Expert Medical Science Safety actions with Agile Groups’ inclusivity and staff adherence to adopt AIDRS developments in an NHSE PHM for us all (NHS England, 2021; NHS England, 2020a). Indeed, global and national reports on conventional neonatal midwifery and primary care practice services should rightfully be considered overwhelmed with real-world cases (Middlemiss et al., 2024; Darzi, 2024). General practice and post-natal infant care receive the HPO system through a GMS safe space ecosystem, which informs workforces about their patients’ rare diseases (1–4) or non-communicable disorders (5–6) from GNBS.

1. Biological ontology’s roles in disease space are valuable targets for detecting disorders from genes spanning mucopolysaccharides, lysosomes, mitochondria, and the urea cycle (GOV.UK, 2021a; GOV.UK, 2023b).
2. Metabolism and pathway processes with anabolic, catabolic, and amphibolic impacts on health outcomes are understood in value early for a midwife through a digital dimension for intervention (GOV.UK, 2021a).
3. Blood cell types with variants that span common haematological conditions to more complex haemoncological diseases while early predictors of abnormalities for GPs personalise plans (GOV.UK, 2021a).
4. Musculoskeletal conditions may be dormant in a dimension, while foresight enables timely paediatric referral for disorders such as achondroplasia, osteogenesis imperfecta, and muscular dystrophy (GOV.UK, 2021a).
5. Physical Health Digital Space informs on major conditions, such as cardiovascular, type 2 diabetes, and chronic respiratory disease, which benefit from plans for personalised intercept (GOV.UK, 2023b).
6. Mental Health Digital Space risk score mental health conditions such as schizophrenia, major depressive disorder, and anxiety disorder as our digital twin presents individual intercepts for quality lives (GOV.UK, 2023b).

In Figure 1, Pre-eXams, eXams, and post-eXams (X = Gen AI channels) pursue health and life science approaches that aim to assist doctors, nurses, and midwives with PHM value to stratify risk and segment pathology to personalise lifestyle or biopharma intercepts with greater efficacy through back-propagation of outcome data (Department of Science, Innovation and Technology, 2023; NHS England, 2021; NHS England, 2020a). The manuscript aims to convey how GNBS provides an understanding of infant biomedical tests and the molecular aspect of haematology disorders, with attention to predictive or diagnostic pre-eXams and biopharma eXams, channelling value-based PHM to staff (Clague and Thomas, 2002; Provan and Lazarus, 2024; NHS England, 2021; NHS England, 2020a). The AIDRS and a proposal for HEMSS with Agile Group developers are conveyed throughout the manuscript to support a workforce on a newborn or citizen HPO.

### 5.3 Cloud hybrid digital system partners

The DSIT system approach must evaluate the aims of a digital economy, considering international benchmarks and market potentials across an ecosystem, to ensure optimal HPO while supporting NHSE PHM and the workforce (Department of Science, Innovation and Technology, 2023; NHS England, 2021; NHS England, 2020a). The PHM ecosystem digital economy and the HPO market potential are exponential in delivering public health and patient safety (Department of Science, Innovation and Technology, 2023). The author considers three Cloud hybrid digital system partners in the PHM GMS safe space, from the least to the most expensive options, and sums them up with HPO biobank aims while sustaining the environment depicted in Figure 1.

The following global partnerships enable genomic data processing and analysis, supporting advancements in research and clinical settings, so presented for the aims of PHM GNBS with multi-omics intercepts.

1. *China*: The MGI DNBSEQ-T20 × 2 sequencer processes rapid WGS for $100 per run to reform PHM with excellent market potential due to the GNBS cost while integrating Microsoft Azure Data Box for secure data transfer and APIs for seamless flow (MGI, 2024).
2. *US:* Google-Broad Institute strategic alliance and partnering solutions store, process, and analyse genomic data with best-in-class analysis tools at scale, with Google Genomics Cloud Platform supporting PHM (Broad Institute, 2016).
3. *UK*: Cambridge University with Illumina partnered with AWS for genomic analysis with HPO publications on secure, cloud-based data for globally accessible infrastructure, with the expected rapid growth of genomic data (Illumina, 2020).

Figure 1 depicts a national approach with the Cloud hybrid digital systems alongside Biobank partners for GNBS and presents risks as agile groups develop for the PHM ecosystem to predict or diagnose health (pre-eXams) or precisely intercept (eXam) for each HPO. Depictions and options 1–3 align cost-effective cloud hybrid digital systems, wherein organisations optimise pricing to facilitate solutions by collaborating on centralised data access and analysis tools with NHSE FDP, an asset for the AISI aim for quantum agentic AI (Independent, 2023; GOV.UK, 2024). These partnerships must also ensure robust infrastructure for AISI safe space and DSIT national PHM security through data transfer, using agile developer methods for AIDRS to propose ICB adoption of pre-eXam-eXams at scale for each point of need (NHS Beta, 2024; GOV.UK, 2024; Institute of Biomedical Sciences, 2023; Department of Science, Innovation and Technology, 2023). Future aims to sustain an ecosystem approach as a PHM mindset will drive HPO System Engineering Initiatives for Public Safety (Carayon et al., 2020) to mitigate NHSE negligence claims through national strategic initiatives (Department of Science, Innovation and Technology, 2023; Dyer, 2023).

## 6 Determine the next-generation predictors in the HPO pre-eXam

In Figure 1, the Pangenome unlocks our HPO system with GNBS pre-eXams that review the validity of traditional infant biomedical and haematology tests in a lifetime (Clague and Thomas, 2002; Provan and Lazarus, 2024). Critical literature reviews of AI in diagnostics scope the next generation of predictors for a timely and accurate digital HPO evaluation (Mirbabaie et al., 2021; Kumar et al., 2022). The key takeaway is that determining the next generation of predictors and diagnostics in the pre-eXam proposal will accelerate HPO with data and personnel training against this plan and an ongoing evaluation (Williams, 2024). Section 6.1 determines the next-generation predictors by pangenome. After that, in Section 6.2, GNBS realigns blood spot biomedical spectrophotometric validations. Section 6.3 GNBS realigns blood cell haematological operational procedures. Section 6.4 GNBS aligns multi-omics in HPO pre-eXams.

### 6.1 Determine the next-generation predictors by pangenome

In Figure 1, the pangenome spans points 1–3 from the NHSE ecosystem (point 1) to the community for each HPO point of need (point 3). In contrast, point 2 refers to the pangenome above in secure cloud hybrid regions for safer PHM while developed through Agile Groups Development for ICB region adoption.

The inclusion of 350 personal nucleotide references enhances risk assessment of dark data introns to HPO disease precursors, representing a subset of a planned panel aimed at capturing diversity across 700 haplotypes (Liao et al., 2023). We utilize a global resource map provided by the pangenome project to advance predictors by pangenome, which is crucial for understanding and addressing complex genetic variations across different populations (New Scientist, 2023; Wang et al., 2022). The pre-eXams information is s extensive and, despite covering rare diseases and non-communicable disorders previously discussed, it offers specificity to target drug doses in pharmacogenomics, optimises nutrients, and provides future direction in ontology (Goodman and Brett, 2021; Cole and Gabbianelli, 2022; Sirén et al., 2024).

With HPO agile group developments such as DRAGEN, the Nature Journal reports comprehensive genome analysis and variant detection at scale, while next-generation predictors by Pangenome require the expertise of medical health and life science professionals to determine requirements and interpret findings (Behera et al., 2024). GNBS’s comprehension of exomes, intronic, intergenic, and regulatory regions enables the assay of single nucleotide variants, small insertions and deletions, repeat expansions, copy number variants, and other structural variants that also explore the impacts of enhancers and promoters (Lin et al., 2019; Li, 2022). The promise of GNBS, using WGS with deep learning within a broader pangenome framework, will deploy unparalleled predictors across all HPO disorders with a genomic predisposition, creating a unique fingerprint for everyone (Tavakoli et al., 2023).

### 6.2 GNBS realigns blood spot biomedical spectrophotometric validations

Figure 1 shows genomes from the Pangenome and introduces a GNBS Pre-eXam risk stratification for rare diseases and major conditions in predictive HPO. The main challenges in newborn biomedical blood spot screening include obtaining high-quality outcome data and integrating diverse data sources, which can lead to inaccurate diagnoses, delayed treatments, and missed opportunities for early intervention (Clague and Thomas, 2002; GOV.UK, 2022). Pre-eXam variant calls for newborn disease, which replicate 10 blood spot screens, were assessed using a Genomic England (GE) PanelAPP and a novel pangenome concept, as presented in Table 1. This approach supports the UK Government and NHS Genomic England initiatives and extends outreach to the US National Human Genome Research Institute (England, 2024; Genomics England, 2023; NHS England, 2023; National Human Genome Research Institute, 2023).

**Table 1:** Pangenome GNBS realign specialist biomedical mass spec analysis.

Biomedical and inherited metabolic diseases for mass spectrometric analysis	GE PanelApp [X = Intelligence search] for new pangenome reference
Phenylketonuria is an autosomal recessive disorder caused by a deficiency of phenylalanine hydroxylase (PAH), leading to high levels of phenylalanine. Early detection and a specialised diet can prevent severe mental retardation and other complications.	Genomics England PanelApp locates 11 panels, 3 red, and 8 green. While the green panels present strong evidence for gene-disease associations, the red panels have scant research evidence and should not be used.
Medium-chain acyl-CoA dehydrogenase deficiency is a rare genetic condition where the body cannot break down medium-chain fatty acids, leading to energy production issues. It causes severe hypoglycaemia and sudden death if not treated.	Genomics England PanelApp Online Mendelian Inheritance of Man number 607008 informs on cloning and expression, gene structure, gene function, mapping, and molecular genetics.
Maple syrup urine disease is an autosomal recessive disorder caused by a branched-chain alpha-keto acid dehydrogenase complex deficiency. It leads to elevated levels of branched-chain amino acids, causing severe neurological damage or death if untreated.	MSUD mutations are in BCKDHA, BCKDHB, BCKDK, DBT, DLD, and PPM1K. These genes appear in other panels rather than a specific MSUD panel with MSUD not located on the Genomic England PanelApp.
Isovaleric acidaemia is a rare inherited condition where the body cannot process leucine, leading to a harmful build-up of isovaleric acid. It can cause severe metabolic crises and brain damage if untreated.	The National Library of Medicine Centre Information: 769 germline mutations, 346 likely benign, 184 uncertain significance, 108 likely pathogenic, and 87 pathogenic.
Glutaric aciduria type 1 is a rare inherited disorder caused by a deficiency of glutaryl-CoA dehydrogenase, leading to a build-up of glutaric acid. It can cause severe brain damage and physical disabilities if untreated.	Comparing gene ENSG00000105607 to the new pangenome reference, the differences include diverse alleles or structural variations to understand gene diversity or disease associations.
Homocystinuria is a rare inherited disorder caused by a cystathionine beta-synthase (CBS) deficiency, leading to high levels of homocysteine and methionine. It can cause severe health issues if untreated.	CBS mutations located in 15 panels have no homocystinuria panels, nor is the disorder term acknowledged in the GE PanelApp. One panel was reported as amber to proceed with caution.
Classical galactosaemia and its pathologies, which are impacted by Empirase and galactose kinase deficiency, require differentiation from the non-symptomatic variants.	Galactose-1-phosphate uridylyltransferase was not located, while genes GALT [12 Panels], GNE [15 Panels], and GALK1 [11 Panels] were located in the GE PanelAPP.
Gaucher disease is an autosomal recessive inherited lysosomal storage disease that occurs in early childhood from the β-glucocerebrosidase deficiency impacting organ failure.	The Genomics England PanelApp refers the public user or practitioner to the Gene2Phenotype, which referred to Disease Types 1–3 and 3C as lethal and provided 93 phenotypes.
Tay-Sachs disease is a neurodegenerative disorder. An autosomal recessively inherited deficiency of β-hexosaminidase progresses to neurological deterioration and early childhood death, with no cure currently available.	The HEXA 20 panels may need to create another panel whereby each dataset focuses on a disease grouping of clinical presentations relevant to Mendelian disease’s clinical diagnosis.
Spinal Muscular Atrophy is a neuromuscular disorder that results in a loss of motor neurones and progressive muscle wasting; it is a common cause of infant death, with no cure currently available.	Spinal Muscular Atrophy type 1 rare PheneGen Spatial and Linear graphs accessed Types with Phenotype MIM and Gen/Locus numbers provided.

Biomedical testing and quality assurance in detecting inherited metabolic diseases for mass spectrometry analysis provide false positives and negatives in newborn blood spot screening, which can significantly impact patient safety by leading to unnecessary additional testing for families with flawed outputs, delaying critical treatment, resulting in severe health consequences and death in some cases (England, 2024). Spectrophotometry delays and sample quality exacerbate risks, as timely blood spot integrity, diagnosis, and intervention are crucial for testing accuracy and managing metabolic disease (England, 2024).

The GE PanelApp creates, stores, and queries virtual gene panels related to human disorders with a crowdsourcing tool for experts to review genes, providing a standard approach to disease associations (Genomics England, 2023). The Genomic Test Directory lists tests from an NHS GMS for approved gene panels in clinical testing to ensure that only genes with a high level of evidence are included for diagnostic purposes (NHS England, 2023). When interoperable and automated, the pangenome approach can significantly reform biomedical practice, improving the accuracy of detecting genetic disorders and mitigating false negatives or positives (England, 2024; Genomics England, 2023; NHS England, 2023; National Human Genome Research Institute, 2023).

### 6.3 GNBS realign blood cell haematological operational procedures

Arcane systems initiate comprehensive testing for conditions such as anaemia, haemostasis, haemophilia, haematinic, haemoglobinopathy, and malignancy, where tests often involve high demand volumes and face challenges related to subjective interpretation, specificity, and sensitivity, with reference ranges that vary across ethnicities (Wians, 2009). The NHSE genetic test deeply integrates with a robust biobank infrastructure and agile processes for GNBS pre-eXam to address disorders of red, white, and platelet cells or the broader HPO disease classification impacting haematology (NHS, 2023; NHS England, 2024; Provan and Lazarus, 2024).

Seamless Variant Call Formats are tabulated for cloud hybrid digital HPO systems, as previously discussed (MGI, 2024; Broad Institute, 2016; Illumina, 2020). Accordingly, Table 2 lists haemoglobinopathies, haemolytic anaemias, and membrane disorders; Table 3 covers B12 and folate deficiency disorders; and Table 4 details thrombocytopenia, neutropenia [cytopenia], and aplasia processes within a GNBS WGS framework (NHS England, 2024). HPO disorders are differentiated and communicated to the Genomic Information System in real time, with priority results promptly directed to a haematologist or potentially reflexed automatically via another ML/AI algorithm in the FDP (Illumina, 2023).

**Table 2:** Haemoglobinopathy, haemolytic anaemias, and red cell membrane disorders.

Disorder	Genomic germline pre-eXam
Hemoglobinopathies	HBB Thal, HBS, HBA1, HBA2, HBC, HBE
Glutathione Synthetase Deficiency	GSS (e.g., c.1430G > A)
G6PD deficiency	G6PD (e.g., c.563C > T)
Pyruvate kinase deficiency	PKLR (e.g., c.1456C > T)
GP isomerase deficiency	GPI (e.g., c.1040G > A)
Hereditary spherocytosis	ANK1 (e.g., c.4278G > A), SPTA1, SPTB
Hereditary poikilocytosis	SPTA1 (e.g., c.4295del), SPTB
Hereditary elliptocytosis	SPTA1 (e.g., c.6531-12C > T), SPTB, EPB41
Hereditary ovalocytosis	SLC4A1 (e.g., codons 400–408 del)

**Table 3:** Megaloblastic anaemia, B12 and folate deficiency related disorders.

Primary gene	Genomic Germline Pre-eXam
MTR (e.g., c.2756A > G) affects the enzyme methionine synthase	
MTRR impacts methionine synthase, leading to vitamin B12 deficiency.	
MMACHC can cause methylmalonic aciduria and homocystinuria.	
GIF can lead to a lack of intrinsic factors, essential for Vitamin B12 absorption.	
MTHFR (e.g., c.677C > T) reduces the enzyme’s activity in folate metabolism.	
The SLC19A1 gene impacts folate transport into cells, leading to folate deficiency.	
The DHFR gene impairs the conversion of dihydrofolate to tetrahydrofolate.	
PCFT leads to folate malabsorption in the intestines.	

**Table 4:** Thrombocytopenia, neutropenia (cytopenia), and aplasia.

Primary gene	Genomic germline Pre-eXam
MYH9 gene affects the production of myosin-9, leading to anomalies and three syndromes	
WAS gene affects platelet size and function, leading to low counts and immune deficiencies.	
Bernard-Soulier Syndrome: GP1BA, GP1BB, or GP9 genes cause a disorder of large platelets.	
Thrombotic Thrombocytopenic Purpura: ADAMTS13 (e.g., c.4143_4144insA).	
SCN: Often caused by the ELANE gene with a severe reduction in neutrophils.	
Cyclic Neutropenia: The ELANE gene is characterised by periodic drops in neutrophil counts.	
SDS: SBDS causes a syndrome that impacts the bone marrow, pancreas, and skeletal system.	
GATA2 deficiency results in a range of haematological abnormalities, including neutropenia.	
Fanconi anaemia leads to BM failure caused by mutations in one of several genes.	

The proposal for an HPO pre-eXam requires greater GPU to scale up and servers to scale out capacity to expedite the differential capability in PHM (Koorts and Rutter, 2021; Aarons et al., 2017). The tables under-represent the depth of mutations and do not relay the haematology manifestation as detailed for the biomedical disorders in Table 1, column 2, which should be available on an NHSE-approved NEQAS safe space provision in the future (Office, 2023). Indeed, Professor B. Bain, a global lead in haematology, reports on more than 33 hereditary thrombocytopenia and over 50 neutropenia traits (Bain, 2021). The author reports that the X in a pre-eXam, specific for the positive variant call, could be explained for user trust as detailed from the national PanelAPP and genomic test directory, which could become a personalised library (Genomics England, 2023; NHS England, 2023).

Complex myelodysplastic syndromes, myeloproliferative disorders, leukaemia, and lymphomas require annotators for HPO system engineering initiatives for patient safety, as haematologists work on complex genomic profiling (Duncavage et al., 2022; TRC of Pathologists, 2021). GNBS agile group development with workforce solutions based on WGS assurances for precision medicine reforms HPO system assessment (The Royal College of Pathologists, 2021; Ebert, 2017; Lee et al., 2023; Kaplun et al., 2023). Indeed, GNBS realigns both biomedical and haematological operations as an HPO evaluation using two methods to assure and communicate hundreds of treatable disorders. The first is a build on traditional CNNs and RNNs to explore strengths in transformers and other LLMs for genomics [X = Gen AI] (Liu et al., 2024; Consens, 2023). Second, re-evaluating genomes in a pre-eXam using a human pangenome reference captures population diversity within a safe space for greater HPO specificity and sensitivity (National Human Genome Research Institute, 2023). Valid GNBS is a predictive and diagnostic pre-eXam that evolves in a diverse pangenome for excellence in genome prediction, diagnosis, intercept, and outcome, providing variants in the reference pangenome to seek other assurances to negate any residual HPO bias (Sirén et al., 2023).

### 6.4 GNBS aligns multi-omics in HPO pre-eXams

In Figure 1, the application of health pre-eXams for eXams intercepts extends across an HPO, emphasising group assessment for personalised adoption, as shown in Figure 1. While X may not provide definitive evidence, it may indicate probability or likelihood, transferrable to the adopter to ensure trustworthiness and transparency in the HPO system claim. This process includes detailing all appropriate authorisations and regulations to meet personalised X points of need (NHS Beta, 2024).

From GNBS, the navigation of multi-omics facilitates the opening of knowledge on HPO systems, translating disease mechanisms into early diagnosis and innovative approaches through rigorous digital validation (Mohr et al., 2024; Hasin et al., 2017). The comprehensive data flow of multi-omics evaluates conditions and involves numerous components that require agile analysis to accurately value any one phenotype (Nature Portfolio, 2024). Thus, GNBS aligns multi-omics HPO pre-eXams to effectively structure knowledge.

In Table 5, genome screens reflexively validate multi-omics pre-eXams, as science and technology identify biological points of HPO need (Institute of Biomedical Sciences, 2023). The right column of Table 5 presents research biological modeling where the proof of concept and fit-for-purpose analytics align to commission omics in the HPO Pre-eXam; without AIDRS approval for adoption, the governance of multi-omics does not fall under the directorship of the national bodies (NHS Beta, 2024). The left column of Table 5 exemplifies multi-omics that reflect from genome screens at specific gene variants, serving as a simplistic validation of multi-omics pre-eXams. Valid X is explainable in terms of the data trained, cloud server AI QA, genomic profiling, clinical trials, and outcomes as applicable, or it remains within the Trusted Research Environment the Trusted Research Environment.

**Table 5:** Human phenotype ontology, reflex multi-omics as part of the Pre-eXam.

Multi-omics pre-eXams (non-AIDRS multi-omics remain research)	HPO pre-eXams are validated (biological modeling)
Pharmacogenomics	How genes influence drug responses
Nutrigenomics	How diet affects the expression of genes
Transcriptomics	How the transcriptome builds from genes
Proteomics	How the proteome builds from genes
Glycomics	How glycoproteins/glycans are impacted by genes
Metabolomics	How genes impact metabolites
Lipidomics	How genes impact lipid metabolism

In Table 5, column 1, multi-omics agile group developers in pharmacogenomic pre-eXams is a competitive market from research to practice in a governance and normative approach to clinical trial development in which the AIDRS approve precise care intercepts as an eXams classification with the application of X, a development that is certified as commissioned for adoption (Nogueiras-Álvarez, 2023; Kabbani et al., 2024).

Table 5 progresses with the application of nutrigenomics, which spans health and disease and integrates other omics that need a holistic ontology approach for a deeper understanding of each human biological system, such as the impact on metabolism (MDPI, 2024; Lagoumintzis and Patrinos, 2023). Transcriptomics and proteomics pre-eXam translations with instances such as gene expression or protein folding aim to target accurate eXams, which detail or track an X developmental process (Tsakiroglou et al., 2023; Jia et al., 2024).

Other disorders from glycomics and glycoprotein are out with the central dogma theory, as research scope evidence-based predictors and precision care intercepts (Mechref and Muddiman, 2016). Building an ecosystem for HPO safe space also digitises our environment to assess impacts on our metabolomics (MDPI, 2023). With dyslipidaemia, the genomics of familial hypercholaesteremia and pharmacogenomic pre-eXams target statin eXams, developing with agile groups for clinical pathway adoption (Chora and Bourbon, 2021).

## 7 Digital regulation services for HPO eXams intercepts

In Figure 1, the Digital Regulation Service (DRS) advances medical science and biopharma HPO risk stratification and commissioning projects such as AHSN-GEP clinical pathway initiatives, which lead pre-eXam and eXam, supporting evidence-based projects for the GMS (Genomics Education, 2024). The research pathways outlined strive for regulatory approval, detailed in the following sections. Section 7.1: Strategise the DRS for HPO personalised plans. Section 7.2: Plan higher expert medical science safety with agile groups. Section 7.3: Research gene, oligonucleotide, and recombinant-protein intercepts. Section 7.4: Commission blood cell eXams and research coagulation intercept eXams. Section 7.5: Monitor predictive health pre-eXam for precise eXam with imaging.

### 7.1 Strategise digital regulation services for HPO personalised plans

Figure 1 outlines a strategy for the AIDRS in the PHM of HPO, detailing the implementation and governance of an ecosystem for genomics and AI technologies. This ecosystem is designed for developers and adopters to engage in efficient and practical projects that predict, diagnose, and intercept diseases. Globally, HPO ecosystems align UK national leads to centralise the GMS HPO system, encompassing both pre-eXams and eXams, facilitating minor stakeholders’ adjustments and adherence. This centralized approach aims to establish structured methods to develop predictive and precision care for adoption (Gargano et al., 2023). Bodies align PHM building HPO process working groups for project management that identify opportunities and value in planning projects with individual plans with an implementation phase in a pilot from research development to the X requirement for national adoption (AI, 2024).

In Figure 1, the AIDRS extends HPO oversight from GMS and biopharma consortiums to implement WGS, multi-omics, and their intercepts as a norm to align sustainable goals and promote wellbeing and growth (United Nations, 2023; UNEP - UN Environment Programme, 2024). The AIDRS provides comprehensive guidance across regulatory, evaluation, and data governance pathways for AI and digital technologies in health and social care (NHS Beta, 2024). Developing Pre-eXam-eXam training for NHS staff while emphasising the importance of public involvement requires education for quick access to treatment by incorporating GMS-AI pathways in a standard HPO manner, building on current AIDRS training programs with real-world X examples (NHS, 2024). Developers and adopters navigate a complex regulatory landscape, ensuring the safe and effective use of projects that embed HPO genomics across NHS primary care with appropriate referrals (Hayward et al., 2023).

AIDRS supports the NIHR and HRA in ensuring that HPO health and social care research meets high standards (NHS Health Research Authority, 2017). The NIHR focuses on funding and supporting health and care research, while the HRA ensures that research is conducted ethically and legally (NHS Health Research Authority, 2017). Their collaboration aims to streamline processes and enhance transparency in HPO research. The AIDRS aids the MHRA in regulating medicines and medical devices, including software as a medical device that impacts HPO, while ensuring that medical products meet safety, quality, and efficacy standards (MHRA, 2019). This collaboration helps the safe and effective use of AI and digital technologies in their application to HPO personalised plans (NHS Health Research Authority, 2017; MHRA, 2019).

AIDRS works with NICE to evaluate the clinical and cost-effectiveness of health technologies to produce evidence-based guidance and advice for health, public health, and social care in an assurance of HPO (NICE, 2021). The partnership ensures that AI and digital technologies are used effectively and provide value for money (NICE, 2021). AIDRS collaborates with the CQC to monitor, inspect, and regulate services while ensuring that services meet clear quality and safety standards for future HPO personalised plans (CQC, 2023). Cooperation helps maintain high standards in using AI and digital technologies, and the AIDRS strategy (NHS Health Research Authority, 2017; MHRA, 2019; NICE, 2021; CQC, 2023), wherein a plan for Higher Expert Medical Science Safety with AIDRS Agile Group Development incorporates pre-eXams and eXams for adoption.

### 7.2 Plan higher expert medical science safety with agile group development

The NHS Long Term Plan aligns the AI Infrastructure Plan to mark an HPO reform in healthcare delivery by the time they conclude in 2029 and 2034, respectively, integrating digital technologies and PHM (Chapman and Middleton, 2019; NHSE, 2024). AIDRS in an ecosystem transition benefits from Higher Expert Medical Science Safety (HEMSS) Agile Group Development for wellbeing with growth in a PHM HPO plan that operates at five levels:

*Level one* is inclusiveness for higher (cloud-hybrid servers), expert (digital AI), medical (practitioners and biopharma flow), science (multi-omics and socioenvironmental themes), and safety (public health, patient safety, and equality) for people the primary stakeholders (van Ede et al., 2023).

*Level two* is engagement with stakeholders for Agile Group Development with gap analysis for agreements across all points of need that plan projects for adoption or the rejection of adoption in using the AIDRS developments, for classical HPO assurances (Institute of Biomedical Sciences, 2023; AI, 2024).

*Level three* is governance and assurance with HEMSS Agile Group Developers operating through regional project pilots under AIDRS authority, NHSE, and GMS directorship with NEQAS to maintain the reform on predictor and intercept with HPO assurances through classifications (AI, 2024; Office, 2023).

*Level four* is classifications, as HEMSS with Agile Group Development formulates the genomic health pre-eXam and predictive care eXam phases for AIDRS developments, which are integrated across ICB and organisational policies while engaging the public as the primary stakeholder on the benefits of adherence (NHS, 2024).

*Level five* is adherence, which applies to levels one to four while adhering to sustainment for wellbeing and economic growth in PHM of HPO by risk stratification and disease segmentation with citizen and practitioner adoption of pre-eXams and practitioner and biopharma adherence to eXams developments (Institute of Biomedical Sciences, 2023; AI, 2024).

Figure 1, HEMSS action with Agile development of HPO knowledge in a child digital health record as a digital twin, details personal health and develops parameters with precise tracking for early identification of potential health issues, thereby enhancing a lifetime of wellbeing.

### 7.3 Research gene, oligonucleotide and recombinant-protein intercepts

Figure 1 aligns digital GNBS from child health to opportune a generation of HPO development as TRE agile group methods develop AIDRS real-world instances. AIDRS validates PHM pre-eXam concepts to ensure fit-for-purpose developments, facilitating the adoption of valid and cost-effective eXams with X explanandum for clinical trustworthiness (Bürger et al., 2024). Consider Section 7.3.1 Gene therapy eXam value and Section 7.3.2 Oligonucleotide and recombinant protein eXam impacts.

#### 7.3.1 Gene therapy eXam value

Global excellence in gene and cell therapy development is a milestone for sustaining good child-to-adult health and wellbeing through experts in a new global age of rare disease intercepts (Schambach et al., 2023; Bueren and Auricchio, 2023). In Table 6, the Gene therapy partnership aims to develop with AIDRS for eXams value that impacts greater wellbeing through point-of-need adoption.

**Table 6:** Gene therapy eXam value at the point of need.

Gene therapy eXams (AIDRS approved) intercept from the Genomics pre-eXam	
CRISP-R	Know the Cas9 technology on genes and treat inherited DNA disorders
Gene Addition	Know the science of functional gene copies to address conditions
RNA Therapy	Know the RNA interference or antisense oligonucleotide technologies
Exon Skipping	Know the science of gene addition to select and skip mutation segments
CART-T	Know the technology to modify T cells to express CAR in oncology therapy
Viral Vector	Know the science of viruses, lentiviruses, or adeno-associated for therapy
TALENs	Know the tech, TAL effector-binding, and FoKI domains to break DNA

CRISPR, Clustered Regulatory Interspaced Short Palindromic Repeats; CART-T, Chimeric Antigen Receptor T Cell; TALENs, Transcription Activator-Like Effector Nucleases; ZFNs, Zinc Finger Nucleases; CAR, Chimeric Antigen Receptors.

Table 6 shows the point of need in the HPO space for safe physical and mental health, from genomic pre-eXam to eXam intercept, which is diverse. Segmentation of beta-thalassaemia or sickle cell, typical in consanguine communities, provides HPO diagnosis for the mitigation of premature death with an accurate eXam intercept (McGann et al., 2017; Temaj et al., 2022). One eXam that offers a quality-of-life value is the Clustered Regularly Interspaced Short Palindromic Repeat (CRISPR) for gene edits to locate nucleotides that modulate expression (Hochstrasser and Nuñez, 2021; Kazi and Biswas, 2021). Other eXams principles for genome editing are longevity applicable in each row of Table 6.

Human gene therapy regulation involves the MHRA overseeing clinical trials and marketing authorisations for gene therapy products, ensuring they meet safety and efficacy standards (Medicines and Healthcare products Regulatory Agency, 2023). The Human Tissue Authority (HTA) and the Health Research Authority (HRA) support the regulatory process, providing additional oversight and ethical review (NHS Health Research Authority, 2018). GTAC is the National Research Ethics Committee for gene therapy clinical research, ensuring the necessary moral and safety standards (Imperial College London, 2018). X approval by an AIDRS for authorised eXams in a structured regulatory ecosystem with ongoing gene therapy advancements enhances HPO efficacy and deployment at scale (AI, 2024).

#### 7.3.2 Action oligonucleotide and recombinant protein eXams

Oligonucleotides eXams treat various diseases by targeting genetic sequences (Lauffer et al., 2024). Nusinersen is an antisense oligonucleotide (ASO) used to treat spinal muscular atrophy by modifying the splicing of the SMN2 gene to increase protein production (Chen et al., 2024). ASO Eteplirsen treats Duchenne Muscular Dystrophy by inducing exon skipping in mRNA processing with dystrophin protein production (Iff et al., 2024). Mipomersen targets the mRNA of apolipoprotein B-100 to lower cholesterol in familial hypercholesterolaemia (Burnett et al., 2012). Patisiran is a small interfering RNA (siRNA) therapeutic that targets the mRNA of transthyretin to reduce amyloid deposits in hereditary amyloidosis (Karimi et al., 2024).

Protein therapy eXams for physical or mental disorders, such as diabetes or Alzheimer’s, use advanced biotech methods, such as recombinant DNA (Vajo et al., 2001; Self and Holtzman, 2023). Monoclonal antibodies, enzymes, and growth factor eXams have X versions to define the steps from gene clone to protein formulation (Quinteros et al., 2017; Hennigan and Lynch, 2021; Mullen and Halsey, 2023). There is HPO value in oligonucleotide and recombinant proteins, which vary in adoption depending on trial outcomes or process advancements requiring trust through AIDRS X approval to scale an NHS adoption at the point of need (Institute of Biomedical Sciences, 2023; AI, 2024).

### 7.4 Commission blood cell, haemostasis, and coagulation intercepts as eXams

An AIDRS development of precise eXams aims to address primary or secondary diseases of blood cells, haemostasis, or coagulation, requiring a pre-eXam to determine pathogenicity. The article provides real-world instances in the following sections:

Section 7.4.1: Examines white, red, and platelet cell eXams, and Section 7.4.2: Focuses on haemostasis and coagulation intercepts for eXam approval.

#### 7.4.1 White, red, and platelet cell eXams

An accurate GNBS prediction or diagnosis of blood cell abnormality is feasible for the best-personalised plans in which HPO risk evaluation by WGS is practical to identify germline, stem cell self-renewal, or progenitor cell genetic mutations that risk pathology (Kohlmann et al., 2013). Consider the PHM of HPO, which addresses multi-omics profiles at a point of need as the white cell disorder lymphoblastic leukaemia is targeted by tyrosine kinase inhibitors or monoclonal antibodies to specific gene mutations and pathways (Cancer Research UK, 2024). Diffuse large B-cell lymphoma, CAR-T cell therapy, and eXams modify a patient’s T-cells to attack cancer cells (Sermer et al., 2020).

The option for precision eXams extends the causation of anaemia and schedules gene therapies for particular haemoglobinopathies; therein, the X can provide information and assurances on clinical trial efficacy and approval authority, as discussed previously with CRISPR therapy (McGann et al., 2017; Temaj et al., 2022; Hochstrasser and Nuñez, 2021; Kazi and Biswas, 2021; Medicines and Healthcare products Regulatory Agency, 2023; NHS Health Research Authority, 2018; Imperial College London, 2018). In other instances, gene editing or therapy for G6PD or pyruvate kinase deficiency restores red cell function, while gene editing in hereditary spherocytosis or elliptocytosis restores the erythrocyte cytoskeleton (Grace, 2023; Garcia et al., 2021; Gallagher et al., 2021; Kim, 2023). AIDRS reforms for genome engineering are robust in the choice of eXams and will robustly assess intercept ideals to reverse thrombocytopenia in inherited Wiskott-Aldrich and Bernard-Soulier syndromes that restore thrombocytes (Immune Deficiency Foundation, 2023; Martinez-Navajas et al., 2023).

#### 7.4.2 Coagulation and haemostasis intercepts

In Figure 1, Agile groups scale safe space to workstream solutions as real-world pathogenic examples use quality data aggregation and training with AI assurance to develop accurate coagulation and haemostasis predictors in pre-eXams to target intercept eXams. GNBS foresees the pathogenicity of HPO early for primary coagulation and accurately intercepts it in Tables 7, 8. In haemostasis, Tables 9, 10 apply digital to regulate personalised intercept futures (Office, 2023; Genomics England, 2023). Consider PHM that predicts primary *pro-coagulation* and *haemostasis* for intercepts as follows:

**Table 7:** Procoagulant variants in selected panels with DOAC intercepts.

Pro-coagulant	HPO pre-eXam* panel classifications	DOAC intercepts	Impersonalised therapy
Leiden F5 (R506Q)	FV Six Panels*	The Agile Group Developers may consider evaluating a digital pharmacogenomic to assess an appropriate anticoagulant dose to a pro-coagulant	Low molecular weight heparin, heparin, and warfarin or Anti fibrinolytic* concentrate
Prothrombin G20210A F2	F2 Six Panels*		
Antithrombin Deficiency SERPINC1 (e.g., c.391C > T)	SERPINC1 Two Panels		
Protein C deficiency PROC (e.g., c.565C > T)	PROC Three Panels*		
Protein S deficiency PROS1 (e.g., c.1045G > A)	PROS1 Three Panels*		

Pro-bleed*panels also.

**Table 8:** Pharmacogenomics of DOAC for dosing.

Anti-coagulation therapy and HPO molecular factor		Pharmacogenomic genes
Dabigatran [Pradaxa]	F IIa	CES1, ABCB1
Rivaroxaban [Xarelto]	Xa	CYP3A4, CYP2J2, ABCB1
Apixaban [Eliquis]	Xa	CYP3A4, ABCB1
Edoxaban [Savaysa, Lixiana]	Xa	ABCB1
Betrixaban [Bevyxxa]	Xa	Not Defined
Warfarin-Coumadin-Jantoven	Vit K antagonist*	CYP2C9, VKORC1, CYP4F2

DOAC preferred over Warfarin dosing*.

**Table 9:** Haemophilia predictors, with eXams, research, and impersonalised intercepts.

HPO pre eXam	Gene therapy eXam	Recombinant factors eXams	Gene therapy research intercepts	Impersonalised intercepts
Haemophilia A-F8 (e.g., c.2209C > T)	Valoctocogene Roxaparvovec	Yes	Ongoing	Plasma factor
Haemophilia B-F9 (e.g., c.316C > T)	Etranacogene Dezaparvovec	Yes	Ongoing	Plasma factor
Haemophilia C-F11 (e.g., c.1331G > A)	None	No	Ongoing	Fresh frozen plasmas

**Table 10:** VWD - HPO pre-eXam, with a personalised eXam and impersonalised intercepts.

Von willebrand disease	HPO pre eXam	Recombinant factors eXams	Impersonalised intercepts
VWD Type 1	Conflicting classifications 84 Benign 134 Likely benign 170 Uncertain significance 682 Likely pathogenic 205 Pathogenic 281	Ongoing	Tranexamic acid desmopressin VWF Concentrate
VWD Type 2		Ongoing	
VWD Type 3		Vonvendi	

Table 7 depicts procoagulant HPO GNBS pre-eXams. Manual access to genes that impact the coagulation pathway identifies procoagulant and pro-bleed* pathogenicity variants, with other variants of that gene non-pathogenic or of unknown significance, which impact manual interpretation and slow real-time response times to an infant point of need (Office, 2023; Genomics England, 2023). In a lifetime, HPO intercepts include DOACS and impersonalised therapy in a search for personalised approaches through regulating digital [Table 7].

Table 8 depicts and recommends the integration of pharmacogenomics for DOACs and less so for warfarin. A DRS would develop safer HPO DOAC dosing through dynamics and kinetics evaluations before considering and integrating the pathological dosing requirements in X (Tseng et al., 2018; Emanuel et al., 2023). Warfarin is impacted by other vitamin K factors, such as diet (Lobban et al., 2009), making dosing more challenging, if not impossible.

Table 9 on haemophilia and Table 10 on von Willebrand disease illustrate the use of HPO pre-eXams to assess pathogenicity from germline mutations. Next-generation infant studies leverage Trusted Research Environments (TREs) and biopharma research to develop intercepts, employing digitally regulated input and output data within a secure AI and bioinformatics framework (Kavianpour et al., 2022). These intercepts include personalized approaches such as gene therapy and recombinant factor eXams, while other research or non-personalised intercepts are made available depending on the specific needs identified (Institute of Biomedical Sciences, 2023).

### 7.5 Monitor predictive health pre-eXam for precise eXam with imaging

In Figure 1, Monitoring HPO predictive and diagnostic pre-eXams and eXams intercept benefits from tissue, blood cell, and blood viscoelastic imagery, depending on the PHM query. The GNBS WGS serves as an HPO personalised space to evaluate disorders while identifying unspecified pathology with digital visions (Stark and Scott, 2023; NHS England, 2024). Sections 7.3 and 7.4 discuss the targeting of intercepts to prevent or reverse multiple disorders, as personalised approaches optimise an HPO that benefits from visual affirmations and monitoring of pathogenicity (Office, 2023; Genomics England, 2023; Tables 1–6). Agile groups develop with aggregated data and annotate for HPO risk stratification and pathology segmentation in clinical diagnostics as stakeholders view the bigger picture for PHM (Murphy et al., 2024).

“In a consensus for harmonisation, CNNs excel in digital morphology by analysing medical images, extracting features, and classifying tissue or blood cells (Mindray, 2024). This enhances diagnostics by identifying abnormalities and evaluating intercept efficacy, such as gene therapy, with high accuracy (Grace, 2023; Garcia et al., 2021; Gallagher et al., 2021; Kim, 2023; Immune Deficiency Foundation, 2023; Martinez-Navajas et al., 2023). AI-driven viscoelasticity aids in diagnosing and monitoring conditions such as pro-bleed or pro-coagulant states while also providing insights into the efficacy of personalised management such as DOAC dosing regimens (Tables 7–10). Generative AI will transform the analysis of complex and multimodal data layers and queries, enabling stakeholders using LLMs to make informed decisions for value-based personalised plans in a PHM ecosystem that sustains harmonisation at the point of need (Institute of Biomedical Sciences, 2023; Sengar et al., 2024).”

## 8 Discusses human phenotype ontology as a system

The PHM of HPO excellence requires HEMSS with Agile Group Development for GNBS to personalise plans in a programme proposal for NHS Genomics England. The author moves forward with predictors and intercepts to debate that the AIDRS and national stakeholders benefit from HPO developments with X (Gen AI) processes as the norm at each point of need. Therefore, Section 8.1 discusses global and national HPO knowledge for newborn health, and Section 8.2 discusses the ethics of genomic screens and the right to family privacy. Sections 8.3 and 8.4 explore the benefits and limitations of genomic pre-eXam and eXam screens, respectively. Section 8.5 outlines a steward foreground for HPO X in a safe space for public health, patient safety, and parity.

### 8.1 Global and national HPO knowledge for newborn health

In Figure 1, digital child health GNBS knowledge is an informatics ecosystem that identifies, understands, and provides evidence of HPO disease for PHM. UK Science and Technology approaches expand research and development in personalised, safe spaces to action wellbeing by building a knowledge-enabled NHS for the future (Department of Science, Innovation and Technology, 2023; NHS England, 2020b). Scaling individual dimensions for each infant HPO system requires genome data analysis to predict health for precise care assurances and meets the recommendations for evidence in PHM screening (GOV.UK, 2014). Indeed, the manuscript brings a standard dynamic infrastructure with pre-eXams in section 6 and eXams in section 7 that succinctly validate HPO predictors and intercepts for NHS Genomics England and the UK government while showcasing international reform.

The World Health Organisation guides nations on science and technology as a pioneering UK Generation Study for GNBS science associates and translates rare diseases and non-communicable disorders into a health and nutrition lifecycle (NHS England, 2024; Duke et al., 2021). As genome intelligence generates HPO reasoning, medicine should reverently engage in infant value-based care from profound WGS knowledge with haplotypes that sustain national HPO transformational stages (Duke et al., 2021; Carayon et al., 2020; Department of Science, Innovation and Technology, 2023). Supporting GNBS studies is a commitment to science and technology (NHS England, 2024; Department of Science, Innovation and Technology, 2023; Carayon et al., 2020). This manuscript’s goal is a PHM ecosystem approach to practising medicine ethically and digitally, with Tables 1–10, which phase HPO scientific excellence in Higher Expert Medical Science Safety with Agile Groups. We commit to the wellbeing of NHSE-GMS-AHSN-GEP clinical pathway initiatives (Duke et al., 2021; Department of Science, Innovation and Technology, 2023; Genomics Education, 2024).

In Figure 1, the author’s mindset for hybrid digital AI for medical practitioners and biopharma with genome and social scientists enhances public health and mitigates patient safety events. HPO phases and classifications predict health in a pre-eXam for precise care eXams in a national System Engineering Initiative for Public Safety (Carayon et al., 2020). HPO knowledge excellence improves at a pace only experts can align and model as introns and epigenomics insight gene regulation in a digital ecosystem (Petrillo, 2023; Han and He, 2016). The challenge to structure digital health depends on how stakeholders view primary care, which the author accepts as a PHM ecosystem to segment disorders, learn, evidence, and enhance HPO outcomes through data (McDonald et al., 2024) with phases developed with the AIDRS for national adoption in X phases. In discussing PHM benefits and limitations to stakeholders, one significant debate remains on whether GNBS are moral, notwithstanding the WHO and UK government aims (Duke et al., 2021; Department of Science, Innovation and Technology, 2023).

### 8.2 Debates the ethics of genomic screens and the right to family privacy

GNBS in the “Next Generation Study” is a test for early diagnosis with privacy and consent in the context of a family agreeing to align an infant’s DNA to an informant digital health record since research also aims to evidence personalised non-communicable disorders (NHS England, 2024; Tutty et al., 2024). Predicting untreatable diseases has sparked ethical debate in an A-Z of more than 7,000 rare diseases (National Organization for Rare Diseases, 2019).

- GNBS may uncover conditions that point to uncertain pathology outcomes (Genomics England, 2023), thereby challenging the delicate balance between family autonomy, informed consent, privacy, and the needs of an infant’s best interests when unable to provide a voice (NHS England, 2024; Tutty et al., 2024; National Organization for Rare Diseases, 2019).
- Critics of GNBS could argue that such diagnoses point to undue anxiety and distress to parents, whilst a pre-eXam does equip families to emotionally prepare an infant’s need for early palliative care to improve the remainder of life (Eskins et al., 2017).
- Any positive diagnosis for a disease requires counselling with mental health labelling stigmatisation when, in fact, that individual may also need assistance from the point of diagnosis harm and benefits that come from lifetime predictors (GOV.UK, 2021b).
- The scientific pre-eXams require a change in expert counselling and support services for optimal life span or end-of-life, whereby families seek knowledge to proactively organise and cherish time with their infant, especially when facing the prospect of a life-limiting condition (Kemper et al., 2019; NICE, 2018).
- An infant’s right to privacy also ensues from the pre-eXam results, which would schedule an intercept as an eXam, where that child treatment is laudable in terms of future legal considerations, which could potentially impact new debates for the adultescent who did not wish a gene therapy (Bart et al., 2023; Horton and Lucassen, 2022).

Prioritising ethical and psychological impact for families and newborns is paramount, while a write-to-know (predict) and how to resolve (intercept) is surreal regarding infant death, mental health stigma, and early adult demise, requiring further debate on HPO learning (Ulph and Bennett, 2022). These challenges resolve when insights from multimodal data impact primary care risk stratification of populace HPO systems as analytics develop to segment pathology for support solutions that evidence improved and quality lives through structured knowledge.

### 8.3 AIDRS benefits and limitations of genomic pre-eXam screens

The programme proposal PHM HEMSS with Agile Development commissions evidence or probability research in practice as classified under pre-eXam or eXam activities, albeit X is Gen AI, as required (Figure 1). The primary proposal is that the AIDRS develop PHM projects with HEMSS inclusiveness, engagement, assurance, governance, and adherence for ICBs to adopt national HPO authority. The programme proposal provides an executive function of AIDRS to inform on HRA-MHRA-NICE-CQC research, regulation, assessment, and governance that support HPO AI reform as primary care. The proposal is accountable to other stakeholders with a discussion on the benefits and the limitations of genomic pre-eXam screen adoptions approved by the AIDRS, as detailed in Table 11.

**Table 11:** AIDRS benefits and limitations to genomic pre-eXam screens.

Benefits of genomic Pre-eXams screen health manage each phenotype ontology	Limitations of genomics pre-eXams screens HEMSS with agile group development
*Public:* Pre-eXams enable early detection of rare and major conditions for timely intercepts to prevent morbidity or mortality, providing families with informed choices and support decisions. Public health parity is achieved through equal access to screens for all of us, promoting PHM and reducing the disease burden in personalised journeys that transform healthcare services.	*Public:* Pre-eXam screens of unknown significance in genetic data can cause family stress while waiting for confirmatory testing, as do false negatives and positives. An ideal of predictors requires support on public perception. HEMSS operates for inclusivity, engagement, governance, assurance, classification, and adherence for the community.
*Healthcare providers:* HPO pre-eXam screens workstream a new era in primary care services with direct specialist care engaged digitally. HPO intelligence stratifies risks for agile grouping that evidence solutions with accuracy and efficiency in homecare and healthcare delivery. Pre-eXams are not restricted to GP services but directed to citizens to intercept burdens at the primary care.	*Healthcare providers:* barriers to reform receipt expert AHSN-GEP clinical initiative pathways. Meanwhile, reliance on AI requires continuous updates and validation to ensure accuracy and reliability in these clinical settings. HEMSS Agile Groups support emotive and intelligent solutions with five action levels, while a People Plan and Human Resources further aid pre-eXam reforms.
*Biobanks and cloud hybrid servers:* Play a crucial role in the pre-eXam ecosystem by providing the infrastructure for agile groups to expedite next-generation sequencing (NGS), data integration and standardisation, cloud computing, computational predictive models, and data privacy and security. A generation study sets the HPO pre-eXam platform in our future health	*Biobanks and cloud hybrid servers:* Face ethical and regulatory challenges, and validation processes for new technologies are time-consuming and require rigorous testing to ensure accuracy and reliability, which delays development and adoption. Public trust and apprehension on data security require HEMSS actions to assist AIDRS pre-eXam adoptions.
*Governance and FDPs:* Bring stakeholders together in pre-eXams by aggregating genomic and other data with health risks in determinant trends, aiding resource re-allocation from insights. PHM, supported by System Engineering Initiatives for Public Safety, drives HPO effectiveness. FDPs provide access to rapid pre-eXam for PHM to shape HPO X in public health.	*Governance and FDPs:* Infrastructure cost and complex maintenance balance adverse event litigation. Governance of general-purpose AI is challenging as FDPs interoperate on national HPO initiatives; HEMSS Agile groups developers. Overcome limitations through robust pilots, AISI evaluation, and AIDRS development.

### 8.4 AIDRS benefits and limitations of genomic eXam screens

The proposal is that the AIDRS develops eXam projects with an executive arm in the Higher Expert Medical Science Safety proposal for stakeholder inclusiveness, engagement, governance, classification, and adherence of Biopharma eXams as developed by Agile Groups for approved HPO intercepts. AIDRS HEMSS with Agile Groups aligns with the AHSN-GEP clinical initiative pathways in an ecosystem that supports the national bodies in NHSE-GMS-NEQAS-HSSIB for a safe space to enhance public health and mitigate patient safety events by phased HPO eXam adoptions. The programme PHM HEMSS with Agile Group Development is accountable to stakeholders and oversees eXam versions as directed by the AIDRS authority. Stakeholder benefits and limitations are provided in Table 12.

**Table 12:** AIDRS benefits and limitations of genomic eXam screens.

Benefits eXams intercepts AI digital regulation service to stakeholders	Limitations of eXams intercepts HEMSS with agile group development
*Public:* The benefits of personalised health based on individual genetic and multi-omics profiles enhance early detection and intervention to improve outcomes. The AIDRS offers effective precision health and social care from real-world data and advanced algorithms in eXams.	*Public:* Complex genome data and a need for advanced tools pose challenges in populace accessibility and understanding. Privacy and security concerns need to be addressed for trust. HEMSS eXams inform on the process for public trust while adapting to individual X comprehension.
*Healthcare providers:* eXams flow in real-time clinical initiative intercepts based on genetic data. An eXam ecosystem for personalised plans mitigates healthcare burdens as the integration of cloud-based platforms and biobanks for practice drives efficient and effective healthcare delivery.	*Healthcare providers:* The interoperability of eXam intercepts may be limited, with the need for training with expertise to use tools to negate workforce challenges. However, neighbourhood clinics and pilot initiatives integrate accurate eXams with Agile Group developers.
*Pharmaceutical companies:* benefit from intercept acceleration of drug discovery and development processes for AIDRS approval of an eXam. Working with the AIDRS group, the HPOs are involved in clinical trials. Agile groups cooperate on the AI biopharma design for precise intercepts to reduce time and cost to market.	*Pharmaceutical companies:* limitations for eXam intercepts are a high investment in developing AI drug platforms. Regulatory landscapes for AI and genomics are still evolving and may challenge adherence and approval. However, attaining eXam adoption is a market growth via agile development for HPO reform.
*Biobanks and cloud hybrid servers:* are flexible for an intercept ecosystem infrastructure, scaling, storing, and managing genetic data for computational analyses. Biobanks collect and preserve biological samples, facilitating research and development for an eXam HPO system driving advancements in precision medicine to support all stakeholders.	*Biobanks and cloud hybrid servers:* They need to manage secure, large-scale HPO data in privacy arrangements with the Data Use and Access Bill. Infrastructure is costly and complex to maintain agile group operations. Security limits accelerate data encryption with AI use in cybersecurity to develop robust services for PHM eXams.

### 8.5 A steward foreground for HPO X in a safe space

In the manuscript introduction, the author provided a background to the FDP and the impacts on primary care that can be realised from GNBS and HPO generation, with a final discussion that values a governance foreground for PHM of HPO X pre-eXams and eXams in Figure 1. The PHM programme is operational with FDP channelling digital predictors and intercepts that promise and implement a pangenome evidence base to sustainable wellbeing and economic growth (Schambach et al., 2023). The UK national strategy for truth at the points of need requires:

- FDP aggregated data highlighting genomic health risks and health determinant trends, aiding resource re-allocation from insights.
- FDP supports research collaborations and digital innovation with data-driven HPO effectiveness to learn from adverse events.
- FDP access to rapid AI as a pre-eXams predictor and eXams intercept in public health and health services as a classification proposal.
- FDP standardised protocols and consistent data sharing to reduce organisation, regional, and practice variation for safer family healthcare.
- HPO X with multimodal data for standard CNN, LLM, and GEN AI towards Agentic AI structures that extend NHS services for citizen welfare and government initiatives.
- HPO X will deliver evidence and probabilistic research initiatives for cooperation in a new area of continuous monitoring and engineering.
- HPO X is for AIDRS development of pre-eXams predictors and eXams intercept in public health, wherein each X is prescriptive for data training and AI assurances.
- HPO X is stewarded in a safe space in an expanding population health ecosystem as HEMSS agile groups engineer reform.

There are minor challenges to valid analytics with pangenome reference for excellent public health and patient safety. The GE panel APP (Genomics England, 2023) links an overdiagnosis with an overexuberant biopharma industry that impacts value-based care for reform. However, the AIDRS-*HEMSS* develops for HPO by development, *inclusiveness, engagement, governance, classification, and adherence* to adoptions of HPO X assured solutions only (Office, 2023).

## 9 Conclusion

A future of digital child health awaits with the Generation Study of Genomic Newborn Screens and a new pangenome for infants as a pre-eXam of a lifecycle with individualised Gen AI. This study provides critical insights that shape proof of concept and offers a dynamic transition to an ecosystem for population health management and evolving discoveries in the latest scientific advancements. A national health constitution for digital HPO service is one of risk stratification that becomes a standard personalised practice, incorporating fit lifecycle in future analytics.

The Generation Study remains at the forefront of genomic medicine, integrating continuous research features with personalised evidence-based practices. This ongoing process extends beyond birth assessment, revisited and refined throughout a citizen’s life, while the research ventures into the darker sides of data, AI, and genomics. Higher Expert Medical Science Safety (HEMSS) governance continues in subsequent studies, developing neighbourhood points of need for “Our Future Health,” ensuring robust quality assurance and a commitment to “first do not harm” through robust AI evaluations.

The regulatory framework for biopharmaceuticals ensures rigorous testing and evaluation from research to approval, with innovation improving health outcomes. “Genomic Newborn Screens and Multi-omics Intercepts” is part of a series proposing a population health management program, emphasising the importance of HPO AI and evidence-based practices in modern healthcare, aligning with international HPO missions for health outcome excellence. The ever-adapting emphasis on genomics and AI for HPO provides for good eXam stewardship of biopharma through the AIDRS developer-adopter eXam process.

From a global health perspective, the integration of FDP for HPO X, facilitated by GNBS, provides substantial benefits. HPO X with FDPs relay health determinants and trends, enabling targeted interventions and efficient resource allocation. UK national HEMSS plan inclusiveness, engagement, governance, classification, and adherence for HPO developed by the AIDRS for adoption across ICBs in England with standardised protocols and data sharing, reducing healthcare disparities. HPO X serves as a national foreground for PHM oversight of robust health infrastructure and strategy for our continuous engineering in the interests of public health, patient safety, and parity.
